# Ultrasound-Assisted Water Extraction of *Mastocarpus stellatus* Carrageenan with Adequate Mechanical and Antiproliferative Properties

**DOI:** 10.3390/md19050280

**Published:** 2021-05-19

**Authors:** Maria Dolores Torres, Noelia Flórez-Fernández, Herminia Dominguez

**Affiliations:** Departamento de Ingeniería Química, Facultad de Ciencias, CINBIO, Campus Ourense, Universidade de Vigo, 32004 Ourense, Spain; matorres@uvigo.es (M.D.T.); noelia.florez@uvigo.es (N.F.-F.)

**Keywords:** Box–Behnken design, hybrid carrageenan, antioxidants, antiproliferative, biopolymer

## Abstract

Ultrasound-assisted water extraction was optimized to recover gelling biopolymers and antioxidant compounds from *Mastocarpus stellatus*. A set of experiments following a Box–Behnken design was proposed to study the influence of extraction time, solid liquid ratio, and ultrasound amplitude on the yield, sulfate content, and thermo-rheological properties (viscoelasticity and gelling temperature) of the carrageenan fraction, as well as the composition (protein and phenolic content) and antiradical capacity of the soluble extracts. Operating at 80 °C and 80 kHz, the models predicted a compromise optimum extraction conditions at ~35 min, solid liquid ratio of ~2 g/100 g, and ultrasound amplitude of ~79%. Under these conditions, 40.3% carrageenan yield was attained and this product presented 46% sulfate and good mechanical properties, a viscoelastic modulus of 741.4 Pa, with the lowest gelling temperatures of 39.4 °C. The carrageenans also exhibited promising antiproliferative properties on selected human cancer cellular lines, A-549, A-2780, HeLa 229, and HT-29 with EC_50_ under 51.9 μg/mL. The dried soluble extract contained 20.4 mg protein/g, 11.3 mg gallic acid eq/g, and the antiradical potency was equivalent to 59 mg Trolox/g.

## 1. Introduction

Carrageenans are linear polysaccharides of D-galactose and 3,6-anhydro-D-galactose found in red seaweeds and commercially used as thickening, gelling, texturing, and stabilizing agents in food, cosmetics, and pharmaceuticals [1]. Carrageenans are classified according to their structure, sulfate content, and gel-forming ability, with iota ι-, kappa κ-, and lambda λ-carrageenans being the most industrially relevant. In seaweed, instead of a pure compound, hybrid carrageenans are found, and κ- and ι- forms in particular are attracting interest as gelling agents [2].

These high molecular weight polymers (up to 5000 kDa, average 200–800 kDa) are approved for food applications, whereas degraded carrageenans (10–20 kDa) are not authorized [3]. However, other interesting activities of these degraded polysaccharides such as antiviral, antitumoral, antibacterial, or immunostimulant have been reported [4,5,6,7,8]. These properties depend on the carrageenan type and structure, sulfation degree and location, molecular weight, and processing methods [9]. The complex structure of sulfated polysaccharides and diversity as well as the broad range of bioactivities have limited their clinical development owing to poor bioavailability and potentially serious adverse effects, such as the anticoagulant properties, which may limit their use in oncology owing to the risk of internal bleeding [9,10]. Low molecular weight highly sulphated carrageenans are the most active against tumors, and oligocarrageenans induce apoptosis in cancer cells and weaken the immune suppressing effects of antitumor drugs [5,11], suggesting their coupled utilization as both adjuvant or carrier in anticancer treatments.

Carrageenans are usually extracted from seaweeds using hot aqueous solution with water or a mild alkaline media, such as potassium or sodium hydroxide [12]. Recent trends to limit the use of chemicals have promoted the development of greener solvents, such as subcritical water extraction alone [13] or with ionic liquids as catalyst [14]. Moreover, the use of methods assisted by microwaves [15] or ultrasound [16], allowing reduced operation time and energy consumption compared with conventional extraction methods, is increasing. However, the biopolymers exhibit lower gel strength and viscosity [14]. *Mastocarpus stellatus* contains carrageenans belonging to different families (κ-, ι-, ν-, μ-), with more than 80% being κ- and ι-type, thus being an interesting source for its predictable carrageenan yield and easier harvesting compared with other carragenophytes [17]. This species is currently harvested for phycocolloid industrial purposes, but it is still underutilized regarding other valuable fractions, such as protein and phenolics [18,19].

Different intensification strategies have been applied to *M. stellatus* to enhance the extraction process performance. In previous studies, microwave heating has been applied as a pretreatment before hot water extraction [20], and as a heating strategy during subcritical water extraction [15] of carrageenan and bioactives in shorter times than with conventional extraction [15,19]. Enzyme-assisted extraction allowed a lower temperature and was used to produce phenolic hydrolysates with angiotensin-I converting enzyme inhibition and antioxidant properties. The κ/ι-hybrid carrageenan was used for the preparation of films with increased sulfated proportion, protein content, and radical scavenging capacity, as well as enhanced mechanical properties [19,21]. No information is available on the ultrasound-assisted extraction of *M. stellatus*, but Youssouf et al. have reported higher extraction yield of carrageenans from *Kappaphycus alvarezii* and *Eucheuma denticulatum* in shorter time than with conventional extraction without affecting the chemical structure [16].

Ultrasonic treatment is a practical method for the degradation of κ-carrageenan, leading to low-molecular-weight oligosaccharides with new functionalities and bioactivities different from those of the starting material [6,22,23,24]. This is an environmentally friendly scalable method that does not require the use of chemicals, saving energy and time compared with depolymerization with chemical or enzymatic methods [9,10]. Moreover, the degradation process is more uniform owing to improved homogeneity and agitation caused by sound waves [6,16]. More recently, adequate selection of the operational conditions has been proposed to modulate the mechanical features of carrageenans [25] and antitumoral properties [7].

The present study aims at optimizing the ultrasound-assisted extraction of both carrageenan and bioactives from *Mastocarpus stellatus*, reaching a compromise solution between extraction yields and mechanical and antioxidant properties. The antiproliferative properties of the carrageenan product obtained under optimal extraction conditions are further assessed against selected human tumoral cells.

## 2. Results and Discussion

### 2.1. Optimization of the Ultrasound-Assisted Water Extraction

#### 2.1.1. Carrageenan Yield and Properties

**Figure 1:**
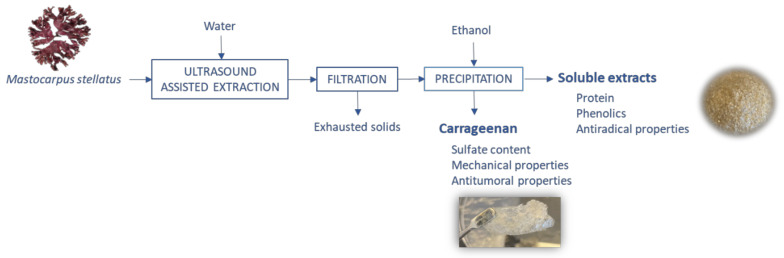
Flow diagram of the ultrasound-assisted extraction of carrageenan and bioactives from *Mastocarpus stellatus*.

The optimization of the ultrasound-assisted extraction of carrageenan and bioactives from *Mastocarpus stellatus* was addressed according to the scheme shown in Figure 1. The influence of the solid to liquid ratio, ultrasound amplitude, and extraction time was studied following a Box–Behken experimental design. After the ultrasound-assisted extraction treatment, the liquid and solid phases were separated by filtration and the solids were discarded. The extracted carrageenan fraction was precipitated with ethanol and was further characterized regarding the sulfate content and the thermo-rheological properties, whereas the remaining soluble fraction was analyzed for phenolic, protein, and antiradical properties. The carrageenan product obtained under optimal operation conditions was evaluated for the antiproliferative activity on selected humal cancer cells.

#### Carrageenan Extraction Yield (CEY)

In order to attain the maximum CEY without jeopardizing the composition and thermo-rheological properties (G′_0_, T_gel_) of the biopolymer as well as the bioactive features of the soluble extracts, the effect of the most relevant operational variables was evaluated. Table 1 summarizes the processing conditions tried according to the proposed experimental matrix and the values corresponding to each response variable. The objective functions were defined based on the calculated regression coefficients summarized in Table 2, which also shows the statistical analysis.

The maximum CEY value (39.5%) was found in the central point of the experimental domain. The lowest CEY was identified at the operation conditions in experiment 1 (SLR: 0.75 g/100 g; A: 75%; t: 15 min). Conventional procedures commonly deliver lower CEY (around 20%) for this type of carragenophyte [26], whereas higher values were attained with microwave-assisted hot water extraction [15], with alkaline solutions in more prolonged times [17] and in a protease-assisted process [21]. The objective function for CEY, described with the coefficients having a significance degree >99%, and expressed in terms of the dimensional independent variables, can be expressed according to Equation (1).

(1) $$CEY_{}=-32.2+20.3\cdot SLR+1.52\cdot t-4.8\cdot SLR^{2}-0.021\cdot t^{2}$$

**Figure 2:**
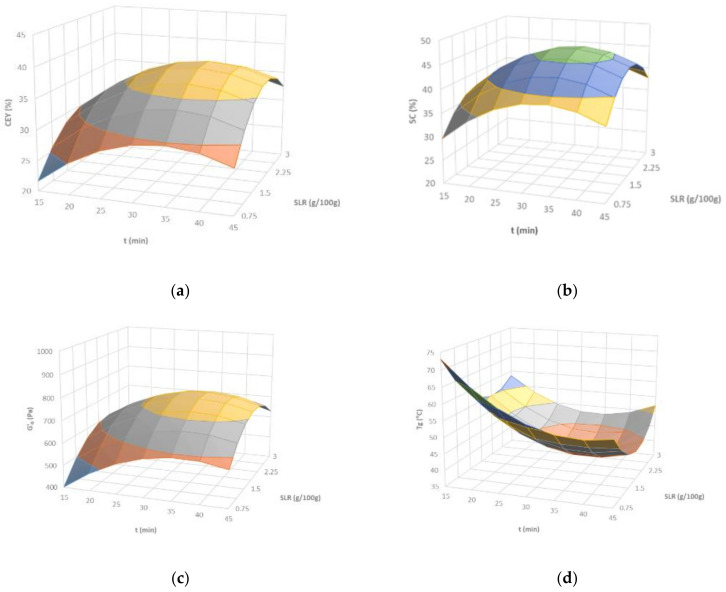
Response surface plots of the (**a**) carrageenan extraction yield (CEY), (**b**) sulfate content (SC), (**c**) G′_0 (1Hz)_, and (**d**) T_gel_, as a function of the solid to liquid ratio and extraction time for a fixed amplitude (75%) during ultrasound-assisted extraction of *M. stellatus*.

The CEY model plotted in Figure 2a shows that, at a fixed time, the solid to liquid ratio (SLR) exerted a more intense effect than amplitude (A). The maximum CEY predicted was 40.3%, within the experimental domain corresponding to intermediate operational conditions (SLR: 2.09 g/100 g; A: 77.7%; t: 35.3 min). When compared with conventional alkaline extraction, these outcomes involved a notable increase in the carrageenan recovery (about twofold) through the reduction of the extraction time (about sixfold) [1]. For the same biological material, conventional hybrid carrageenan production treatment involved a longer extraction time (about 2 h), higher temperature (90 °C), and lower solid liquid ratio (1.5 g/100 g) to deliver a notably lower extraction yield (23.5%) (unpublished data).

#### Sulfate Content (SC)

Maximum values for experiments corresponded to the central point SLR: 1.88 g/100 g, A: 75%, and t: 30 min, followed by the values attained in experiment 6 at 1.88 g/100 g, amplitude 50%, and 45 min. Data in Table 1 confirm a parallel behaviour to the carrageenan extraction yield, and the objective function can be expressed according to Equation (2):

(2) $$SC=-29.8+1.90\cdot t+21.6\cdot SLR-0.026\cdot t^{2}-5.5\cdot SLR^{2}$$

Figure 2b shows the response surface for the sulfate content in carrageenan, with a maximum value predicted at 2.25 g/100 g, operating at 75% over 30 min. This trend suggests that increasing the process severity certain degradation can occur, causing both the loss of recoverable biopolymer and sulfate substituents inducing desulfation. Groult and coauthors reported a more intense desulfation from the native λ-carrageenans during depolymerization with a radical hydrolysis method with H_2_O_2_, from the three sulfate groups per disaccharide in the original to a mean of 0.87 in the final product [10].

#### Thermo-Rheological Features for the Biopolymer-Based Hydrogel Matrices

**Figure 3:**
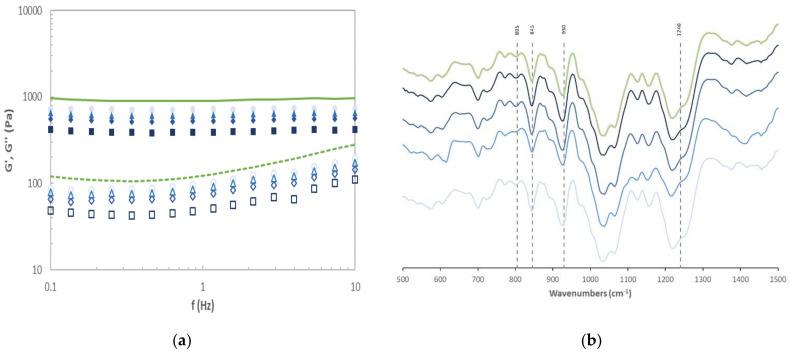
(**a**) Mechanical spectra (G′ (closed symbols) and G″ (open symbols)) for gelled matrixes formulated with representative hybrid carrageenans obtained from ultrasound-assisted extraction of *M. stellatus* in experiments 1 (squares), 5 (diamonds), 10 (triangles), and 15 (circles); (**b**) FTIR-ATR profile for representative carrageenans: 1, 5, 10, and 15 (from dark to light blue). The hybrid carrageenan extracted using an alkali conventional procedure was included for comparative purposes (grey symbols).

The rheological profiles at 25 °C of the κ-/ι- hybrid carrageenans are shown in Figure 3a. All samples exhibited characteristic gel behavior, with the elastic modulus (G′) larger (about 10-fold) than the viscous one (G″) and both viscoelastic moduli almost frequency independent [27]. Magnitudes of G′ and G″ moduli of formulated hydrogels indicated intermediate strength. The experimental viscoelastic properties of formulated hybrid-based hydrogels, expressed in terms of G′_0_, increased with the tested variables up to a maximum of around 725 Pa. These values agree with the results achieved in experiments 13–15 (SLR: 1.88 g/100 g; A: 75%; t: 30 min), followed by those in experiment 8 (SLR: 1.88 g/100 g; A: 100%; t: 45 min). The viscoelastic parameter (G′_0_) for the hybrid carrageenan hydrogels from *M. stellatus* can be calculated at the 99% confidence level by Equation (3):

(3) $${G^{\prime }}_{0}=-663.4+454.9\cdot SLR+9.7\cdot A+31\cdot t-1.33\cdot SLR\cdot t-92.3\cdot SLR^{2}-0.05\cdot A^{2}-0.39\cdot t^{2}$$

Considering the magnitudes of the model coefficients (Table 2), it can be stated that SLR, followed to a lesser extent by extraction time, were the most dominant effects. The linear and quadratic effects of SLR were influential for the viscoelastic features of hydrogels formulated from tested algae, whereas the impact of the extraction time derived principally from the linear terms. The maximum viscoelastic values for hybrid carrageenan gels from *M. stellatus* (G′_0_~742 Pa) were again predicted for intermediate operational conditions (SLR: 2.09 g/100 g; A: 81.3%; t: 34.4 min). It should be highlighted that the optimized ultrasound treatment to jointly achieve the highest CEY and viscoelastic moduli was estimated involving a slight drop in the amplitude (<3% of the above values). These results suggest that the optimized ultrasound treatment enables improving the hybrid carrageenan extraction without jeopardizing the gel strength found using alkaline conventional procedures [1,28].

A clear effect of the extraction conditions is presented throughout G′_0 (1 Hz)_ and T_gel_ (Figure 2c,d). The gelling temperatures significantly varied between 40 and 72 °C depending on the operational conditions, which could be dramatically relevant from the industrial processing point of view. Moreover, higher gelling temperatures were related with softer gelling properties (lower G′_0_ values). Treatments involving higher biopolymer yields also implied enhanced mechanical features. The viscoelastic characteristics of these gels were consistent with those previously found for hybrid carrageenans isolated using conventional treatments for more prolonged extraction times [28] and higher gelling temperatures [29].

Hybrid carrageenans with the lowest gelling temperatures (T_g_) corresponded to experiments 13–15 (SLR: 1.875 g/100 g; A: 75%; t: 30 min), whereas the highest gelling temperatures were identified in experiment 1 (SLR: 0.75 g/100 g; A: 75%; t: 15 min) followed by experiment 3 (SLR: 3 g/100 g; A: 75%; t: 15 min). The predicted equation for gelling temperatures is as follows:

(4) $$T_{g}=156.9-38.1\cdot SLR-0.68\cdot A-2.88\cdot t+0.18\cdot SLR\cdot t+7.51\cdot SLR^{2}+0.033\cdot t^{2}$$

Figure 2d displays the model predictions for gelling temperatures (Tg) considering SLR and extraction time as the independent variables causing the highest effects on the gelling behavior, which is consistent with the results aforementioned for the viscoelastic properties of the hydrogels. Amplitude provoked effects of lower intensity over the studied ultrasounds operational conditions. Namely, the minimum Tg predicted by the empirical model within the operational domain (39.4 °C) was attained with intermediate conditions (SLR: 2.06 g/100 g; A: 81.8%; t: 35.3 min).

The combined optimization of the ultrasound processing attained the strongest gels at the lowest gelling temperatures of the hybrid carrageenans with the highest CEY predicted at SLR 2.08 g/100 g, amplitude 79%, and 34.6 min. As the Tg is critically relevant in specific pharmaceutical, cosmetic, or food applications [29], the control of the processing conditions allowed to deliver a set of gelling hybrid carrageenans, with gelling temperatures varying from 40 to 75 °C, thus satisfying a wide application spectrum. Different features can be determinant on these properties, including the average molecular weight and polydispersity [30], and both conventional and ultrasonic processing can alter the molecular weight of carrageenan [6]. The effect of ultrasound on depolymerization is well known; an example is the work by Tecson and coauthors, who observed a decrease in molecular weight of refined κ-carrageenan, attributable to the hydrolytic cleavage and scission along the chain, especially at the O-glycosidic bond [6].

#### FTIR-ATR

Figure 3b displays representative FTIR-ATR spectra of the ultrasounds extracted hybrid carrageenan. The profiles exhibited the typical iota (at 805 cm^−1^) and kappa (at 845 cm^−1^) bands, which are usually maintained after ultrasound exposure [6]. The infrared absorption band at 930 cm^−1^ suggests symmetric stretching of the (C-O-C) anhidro group, in addition to a broad band at 1240 cm^−1^ attributed to the S = O stretching vibration of the sulphated groups [31,32]. The intensity ratio of infrared absorption bands at 805 and 845 cm^−1^ revealed that the κ/ι hybridization ratio was 0.76 ± 0.02, in the range (75–80% kappa-carrageenan) found for hybrid carrageenans extracted with conventional alkaline treatments [1]. The observed trend is coincident with the data in Table 1.

### 2.1.2. Composition and Antiradical Properties in the Soluble Extracts

#### Protein Content (PC)

Concerning other components and bioactive features of the soluble extracts, the protein content varied over the range from 14.1 to 20.0 mg/g, depending on the ultrasound treatment (Table 1). Soluble extracts with the highest protein content (PC) (19.1–20 mg/g) were determined in experiments 13–15 (SLR: 1.88 g/100 g; A: 75%; t: 30 min), 8 (SLR: 1.88 g/100 g; A: 100%; t: 45 min), and 6 (SLR: 1.88 g/100 g; A: 50%; t: 45 min). This tendency is consistent with the viscoelastic features (G′_0_) of the corresponding hybrid carrageenan hydrogels, as a relevant factor closely associated with the gel strength is the protein content [33]. It should be noteworthy that the best PC extracts obtained in this work using ultrasound treatments exhibited a higher protein content (>7%) than those reported for these species using conventional procedures [34].

The model was less significant than that for other objective functions, with the major effect being observed for SLR (Table 2). Optimal operational conditions predicted for PC of the soluble extracts of *M. stellatus* (20.3 mg/g) were found at intermediate values of the independent variables (SLR: 2.07 g/100 g; A: 80.3%; t: 35.9 min).

#### Total Phenolic Content (TPC) and Antiradical Properties (TEAC)

A slight influence of the extraction conditions on the TPC and TEAC value was identified (Table 1). The maximum experimental values (11.01 mg/g) corresponded to experiments 13–15 (SLR: 1.88 g/100 g; A: 75%; t: 30 min), and were almost double the data obtained under the conditions of experiment 1 (SLR: 0.75 g/100 g; A: 75%; t: 15 min). In general, these contents were higher than those found for other carrageenophytes using conventional alkaline/acid treatments [35,36], and consistent with those reported for *M. stellatus* using microwave-assisted extraction [15]. The empirical equations derived from TPC at a 99% confidence level are given by Equation (5):

(5) $$TPC_{}=-3.84+4.94\cdot SLR+0.32\cdot t-1.01\cdot SLR^{2}-0.004\cdot t^{2}$$

**Figure 4:**
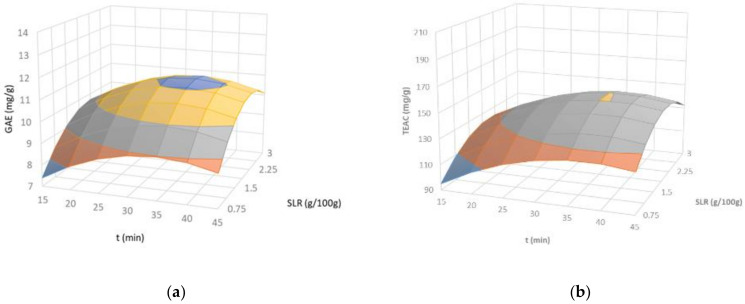
Response surface plots of the (**a**) total phenolic content (TPC) and (**b**) Trolox equivalents antioxidant capacity (TEAC) as a function of the solid to liquid ratio and extraction time for a fixed amplitude (75%) during ultrasound assisted extraction of *Mastocarpus stellatus*.

The response surfaces for tested red algae describing the dependence of TPC on the designated independent variables are displayed in Figure 4a. The major contributions to the TPC on the soluble extracts were identified for the SLR and t, achieving maximal predicted values about 11.3 mg/g at intermediate operational conditions (SLR: 2.22 g/100 g; A: 82.3%; t: 35.6 min). Note here that the TPC dropped at the highest contact time, which suggests that prolonged extraction time at higher temperature (80 °C) can cause certain thermal degradation of these compounds. This behavior is consistent with the results previously found during conventional solvent extraction of phenolics from a number of seaweeds [3]. The maximum antiradical capacity for soluble extracts (~155 mg/g) was obtained under conditions of experiments 13–15 (SLR: 1.88 g/100 g; A: 75%; t: 30 min). These trials also provided the soluble extracts with the largest TPC, suggesting that the phenolic fraction could be responsible for this activity [3]. The dependence of the TEAC values of soluble extracts recovered from *M. stellatus* can be expressed as follows:

(6) $$TEAC=-74.4+72.3\cdot SLR+1.53\cdot A+5.03\cdot t-15.2\cdot SLR^{2}-0.059\cdot t^{2}$$

The above equations were significant at the 99% confidence level, with the most influential independent variable being the SLR, followed by the double effect of this variable, extraction time, and amplitude. Representative impacts of the dominant variables on the TEAC values are illustrated in Figure 4b. The predicted optimal conditions for the antioxidant capacity (159.3 mg/g) were obtained at SLR: 2.11 g/100 g, A: 79.3%, and t: 36.5 min.

Analyzing the combined effects, the proposed model indicated that, for an integral valorization of this seaweed, the optimal operational conditions should be defined by contact times of 34.7 min, SLR of 2.10 g/100 g, and A of 79.0%. These conditions would allow to achieve maximum GAE (11.3 mg/g) and TEAC (159 mg/g) in the carrageenan-free liquid, without jeopardizing the CEY (40.3%), G′_0_ (741.4 Pa) and with the lowest Tg (39.4 °C).

### 2.2. Growth Inhibition of Human Cancer Cells

**Figure 5:**
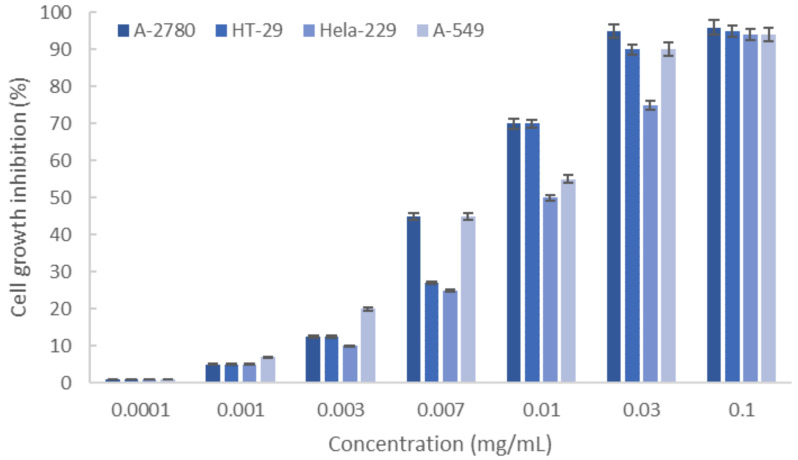
Cell growth inhibition of different cancer cell lines (A-2780, HT-29, HeLa 229, and A-549) caused by κ-/ι- carrageenan extracted from *M. stellatus* under selected conditions.

The antiproliferative effect of the carrageenans recovered from *M. stellatus* after ultrasound-assisted extraction under the optimal extraction conditions on selected human cancer cells is shown in Figure 5. The extracted hybrid carrageenan caused growth inhibition higher than 91% on ovarian (A-2780), lung (A-549), colon (HT-29), and cervix (HeLa-229) carcinoma cells. The lowest IC_50_ values were identified for A-2780 and for A-549 cells, with average values of about 7.2 and 7.5 μg/mL, respectively. The EC_50_ concentrations for the control drug Cisplatin for the tested cell lines were in the range of 0.39–7.46 μg/mL (Table 3). The values obtained for the ultrasound extracts are in the range of those previously reported for native, commercial, and depolymerized carrageenans [7]. A comparative summary of the IC_50_ values obtained for *M. stellatus* in this work and those reported for different carrageenan types against human tumoral cells is shown in Table 3.

The present results were more favourable than those found for the conventionally alkaline extracted carrageenan, i.e., against the A-2780 cells, maximum inhibition of 8% was attained and the EC_50_ was 689 μg/mL (unpublished data). Similarly, *H. musciformis* hydrolysates obtained by papain digestion showed inhibition under 50% for SH-SYS5 neuroblastoma cells and MCF-7 breast cancer cells [37], and both commercial and native λ-carrageenan only slightly affected the cell viability in mice melanoma B16-F10 and breast cancer 4T1 cells [4]. However, slightly higher activity has been reported for the disaccharide κ-carrabiose against A-549 cells (IC_50_: 30–99 μg/mL), being more potent than oligosaccharides in a panel of human and murine cancer cells [5]. As a general trend, the depolymerized carrageenans showed higher antiproliferative action than native or unrefined ones [7,38], but a number of factors, including the carrageenan type, molecular weight, sulfate content, and processing techniques, have also been suggested as key factors. However, much research is still needed for conclusive recommendations, particularly regarding the sulfate content and location [3,5,7].

In this study, an initial test in representative tumoral cells was performed, but further evaluation should be carried out on non-cancer cells and on other cancer lines, such as those from the gastrointestinal tract, which could be a likely route for orally administration [9]. Other activities should be evaluated, for example, anticoagulant properties typical of higher molecular weight compounds, which could negatively affect the therapeutic applications [10].

## 3. Materials and Methods

### 3.1. Materials

*Mastocarpus stellatus* (moisture content of 8.9 ± 0.6 g/100 g) was provided by Compañía Española de Algas Marinas S.A., CEAMSA (Pontevedra, Spain). Seaweeds were stored in darkness in sealed plastic bags at room temperature until further use.

### 3.2. Ultrasound-Assisted Extraction

Ground seaweed samples with average particle size <1 mm were placed in contact with tap water for the desired time at the selected solid to liquid ratio (SLR) in capped Erlenmeyer flasks. Ultrasound-assisted extraction was conducted in an ultrasonic bath (FB11207, 1130 W, Fisherbrand Scientific, Ottawa, Ontario, Canada). Operation was fixed at 80 kHz and 80 °C, as milder conditions tested in preliminary studies did not provide noticeable effects.

Response surface modelling was used to determine the optimum conditions for the extraction of both carrageenans and antioxidant compounds from *M. stellatus*. A Box–Behnken design was chosen to evaluate the influence of the solid–liquid ratio (SLR, 0.75–3 g/100 g), amplitude (A, 50–100%), and extraction time (t, 15–45 min) (Table 1). The objective functions defined to assess the biopolymer extraction were the carrageenan extraction yield (CEY, %), the sulfate content (SC, % wt), the viscoelastic features of the corresponding gels (G′_0 (1 Hz)_, Pa), and the gelling temperature (T_gel_, °C). Those defined to evaluate the composition and antiradical properties of the recovered liquid phases were the protein content (PC, mg/g extract), total phenolic content (mg GAE (gallic acid equivalents)/g extract), and TEAC values (mg Trolox equivalents/g extract). The experimental matrix consisted of 15 combinations, including three replicates at the center point (Table 1). The objective or response functions (Y) were composed of linear, quadratic, and interaction components:

(7) $$Y_{i}=a_{0}+a_{1}X_{1}+a_{2}X_{2}+a_{3}X_{3}+a_{4}X_{1}X_{2}+a_{5}X_{1}X_{3}+a_{6}X_{2}X_{3}+a_{7}X_{1}{}^{2}+a_{8}X_{2}{}^{2}+a_{9}X_{3}{}^{2}$$

where a_0_ represents the model intercept; a_2_–a_3_ are the coefficients of the linear effects; a_4_–a_6_ are the coefficients of the interaction effects; a_7_–a_9_ represent the coefficients of the quadratic effects; and X_1–3_ are the above coded independent variables.

For each experiment, phase separation was accomplished by centrifugation at 1512× *g* for 10 min. Carrageenan fractions were precipitated from the liquid phases with 96% ethanol (1.5 volumes), obtaining carrageenan-free liquid phases (labelled as soluble extracts) and carrageenan fractions. In order to recover the carrageenan, the precipitates were vacuum filtered, washed twice with 96% ethanol, and air dried at 40 °C for 24 h in a convective oven. The ethanol present in the soluble extracts was removed using a rotary evaporator prior to the corresponding characterization. Both soluble extracts and carrageenans from *M. stellatus* were cold stored at 4 °C until further analysis.

### 3.3. Analytical Methods

#### 3.3.1. Extraction Yield

The carrageenan extraction yield (CEY) was gravimetrically determined as previously reported [1].

#### 3.3.2. Soluble Sulphate Content

The soluble sulphate content was determined by the gelatine-barium chloride method described previously [41]. In order to prepare the gelatin-BaCl_2_ reagent, 0.5 g gelatine powder (Scharlau, Madrid, Spain) was dissolved in 100 mL at 70 °C. When the gelatin solution reaches room temperature, it must remain at 4 °C overnight. After this time, 0.5 g BaCl_2_ (Sigma-Aldrich, St. Louis, MO, USA) was incorporated, obtaining a cloudy solution. After 2–3 h, the reagent was ready to be used. Carrageenan extracts or blank (0.1 mL), TCA (trichloroacetic acid) solution at 4% (1.9 mL), and gelatine-BaCl_2_ reagent (0.5 mL) were mixed. The suspensions were incubated for 15 min at room temperature prior to the absorbance reading (500 nm).

#### 3.3.3. Protein Content

The protein content (PC) of the carrageenan-free liquid phases was spectroscopically determined [42]. Briefly, 200 µL of Bradford reagent was diluted with 790 µL distilled water and mixed with 10 µL soluble extracts or bovine serum albumin (BSA, Sigma, St. Louis, MO, USA). The absorbance was read at 595 nm after 5 min of incubation at room temperature. Measurements were performed at least in triplicate.

#### 3.3.4. Total Phenolic Content

The gallic acid content (GAE) was determined according to the Folin Ciocalteu method [43]. One milliliter of the soluble extracts was mixed with 1 mL Folin–Ciocalteu reagent and with 2 mL of a 20% sodium carbonate solution. After 45 min incubation, the absorbance was read at 730 nm, and the results were calculated as gallic acid (Sigma, St. Louis, MO, USA) equivalents. Experiments were made at least in triplicate.

#### 3.3.5. Trolox Equivalent Antioxidant Capacity

ABTS radical cation (ABTS^+^) was produced according to the method previously described [44]. Briefly, 1 mL ABTS was mixed with 10 µL of the soluble extracts or Trolox in a water bath at 30 °C. After six minutes, the absorbance was measured at 734 nm. Measurements were run at least in triplicate, and the results were compared to those of Trolox (Sigma, St. Louis, MO, USA) and expressed as Trolox equivalents antioxidant capacity (TEAC).

#### 3.3.6. FTIR-ATR

The carrageenan fractions were analyzed by Fourier transform infrared attenuated total reflectance (FTIR-ATR) for a qualitative description. These measurements were performed on a Nicolet 6700 spectrometer (Fisherbrand Scientific, Ottawa, ON, Canada) from 500 to 1500 cm^−1^ at room temperature.

#### Thermo-Rheological Testing

Aqueous carrageenan solutions were formulated with 1.0 *w*/*w* biopolymer content and 0.1 mol/L potassium chloride, selected based on previous studies for conventionally extracted products [1,45]. The solutions were prepared by dissolving the carrageenan at 80 °C under strong stirring to ensure full dissolution. The viscoelastic behavior was evaluated in terms of storage (G′) and loss (G″) moduli during the gel forming and was performed on a controlled-stress rheometer (MCR 302, Anton Paar, Graz, Austria) by means of small amplitude oscillatory shear testing. The biopolymer solutions, placed on the pre-heated (80 °C) sand blasted parallel plates (1 mm gap, 25 mm diameter), were sealed with paraffin oil to prevent evaporation, and equilibrated for 5 min at 25 °C. First, stress sweeps were performed varying the shear stress from 0.1 to 100 Pa at 1 Hz and 25–80 °C to determine the linear viscoelastic region for aqueous carrageenan solutions (0.1–18 Pa) and for the gelled matrices (0.1–45 Pa). Second, the rheological testing consisted of (i) cooling temperature sweeps from 80 to 25 °C (1 °C/min, 1 Hz, 10 Pa) to monitor the viscoelastic behaviour and to define the gelling temperature, T_gel_ (tan δ = G″/G′ = 1); (ii) time sweeps (15 min, 1 Hz, 15 Pa, 25 °C) to determine the gel maturation kinetics; and (iii) frequency sweeps from 0.1 to 10 Hz (15 Pa, 25 °C) to determine G′_0_ (1 Hz).

### 3.4. Antiproliferative Activity

Hybrid carrageenans from *M. stellatus* obtained under the optimal extraction conditions were assessed for growth inhibition of four humal cell lines. Lung carcinoma cells (A-549) were cultured on DMEM (Dulbeco modified Eagle’s medium-low glucose) supplemented with 10% FBS (fetal bovine serum) and 2 mM L-glutamine. Ovarian carcinoma cells (A-2780) were cultured on RPMI (Roswell Park Memorial Institute) culture medium supplemented with 10% FBS and 2 mM L-glutamin. Cervix carcinoma cells (HeLa-229) were cultured in DMEM complemented with FBS and L-glutamine at the same proportions indicated above. Colon carcinoma cells (HT-29) were cultured with McCoy’s 5a medium modified growth medium supplemented with 10% FBS and penicillin (100 unit/mL)/streptomycin (100 μg/mL). Cells were incubated at 37 °C under 95% air (5% CO_2_) atmosphere.

Cell growth inhibition was determined with the 3-[4,5-dimethylthiazol-2-yl] -2,-5 diphenyltretrazolium bromide (MTT) method, when transformed to formazan by viable cells. Incubation of A-549 cells was made with a density of 5000 cells/well for 24 h. For A-2780, cells were seeded with a density of 4000 cells/well in 100 µL medium and were incubated for 24 h, then the samples dissolved in water were added and incubated for 96 h. HeLa 229 cells’ incubation was carried out with 4000 cells/well during 4–6 h and, afterwards, the samples dissolved in water were incorporated and incubated over 48 h. For HT-29 cells, a density of 10,000 cells/well was employed and the incubation was performed for 24 h. Subsequently, the carrageenans dissolved in water were added and incubated for 72 h.

After cell incubation at 37 °C under 95% air/5% CO_2_ atmosphere, 10 μL of a 5 mg/mL MTT solution in PBS was incorporated into each well. The mixture was incubated over 4 h and 100 µL of a 10% SDS solution in 0.01 M HCl was added. The mixture was incubated over 12–14 h and absorbance was read at 595 nm (Tecan Infinite M1000 Pro) at least in triplicate. The growth Inhibition percentage was determined according to Equation (8):

(8) $$Inhibition\;(\%)=100-\left(\frac{AO}{AT}100\right)$$

where AO and AT are the absorbance of the sample and water, respectively.

The Inhibitory potential was calculated based on the following equation:

(9) $$Inhibition\;\left(\%\right)=\frac{E_{max}}{1+{\left(\frac{IC_{50}}{EConc}\right)}^{n}}$$

Whenever the Inhibition percentage is plotted versus the sample content (E_Conc_), the maximum inhibitory effect is E_max_, the content inhibiting growth by 50% is IC_50_, and *n* is the slope.

### 3.5. Statistical Analysis

Statistical treatments were conducted using a one-factor analysis of variance (ANOVA) using Minitab 19 software (Minitab LCC, State College, Penn, USA). The impact and regression coefficients of individual linear, quadratic, and interactive terms of the Box–Behnken experimental design were determined. The significances of all terms in the polynomial were statistically assessed by computing the F-value with 99% confidence (*p* < 0.05).

## 4. Conclusions

The Box–Behnken design was useful for the optimization of ultrasound-assisted treatment of *Mastocarpus stellatus.* The solid liquid ratio and contact time were the operational conditions with the higher contribution to the process, whereas limited effects were associated with the ultrasound amplitude, probably owing to the high operating frequency. Intermediate operational conditions with extraction times of ~35 min, solid liquid ratios of ~2 g/100 g, and ultrasound amplitude of ~79% were the best to produce hybrid carrageenans with suitable thermo-mechanical properties without jeopardizing the antioxidant characteristics of the soluble extracts. The properties were remarkably improved compared with those achieved during conventional treatments and the hybrid carrageenans were cytotoxic against four human carcinoma cell lines (A-549; A-2780; HeLa 229), featuring IC_50_ < 51.9 µg/L. Overall, the proposed eco-friendly ultrasound treatment is a simple and flexible method achieving extraction and depolymerization in short times and offering an integral valorization of *M. stellatus* that could be extensible to other carrageenophytes.

## Figures and Tables

**Table 1:** Box–Behnken experimental matrix for the ultrasound-assisted extraction of carrageenan and bioactives from *Mastocarpus stellatus*, expressed in terms of dimensional and dimensionless independent variables, and corresponding experimental values of the response functions: carrageenan extraction yield (CEY), sulfate content (SC), elastic module (G′_0 (1 Hz)_), and gelation temperature (T_gel_) of the carrageenan fraction; protein content (PC), total phenolic content (TPC), and ABTS radical scavenging capacity (Trolox equivalents antioxidant capacity, TEAC) in the liquid phase.

Exp.			Variables (Coded Levels)				Response Functions			
	SLR, g/100 g (X_1_)	A, % (X_2_)	t, min (X_3_)	CEY, %	SC, %	G′_0 (1 Hz)_, Pa	T_gel_, °C	PC, %	TPC, mg GAE/g	TEAC, mg/g
1	0.75 (−1)	75 (0)	15 (−1)	23.7	29.4	392	72	14.1	7.5	101
2	0.75 (−1)	75 (0)	45 (1)	30.1	35.8	578	52	17.7	9.7	134
3	3.00 (1)	75 (0)	15 (−1)	26.9	30.5	498	59	15.9	8.2	114
4	3.00 (1)	75 (0)	45 (1)	34.1	37.9	604	50	18.1	10.1	141
5	1.88 (0)	50 (−1)	15 (−1)	28.1	36.8	525	58	16.3	8.6	120
6	1.88 (0)	50 (−1)	45 (1)	35.2	44.7	650	47	19.1	10.7	149
7	1.88 (0)	100 (1)	15 (−1)	30.3	29.6	570	53	17.5	9.4	131
8	1.88 (0)	100 (1)	45 (1)	36.1	34.3	672	45	19.3	10.8	150
9	0.75 (−1)	50 (−1)	30 (0)	27.5	38.6	481	59	15.8	8.1	112
10	3.00 (1)	50 (−1)	30 (0)	33.4	41.2	623	49	18.4	10.2	143
11	0.75 (−1)	100 (1)	30 (0)	29.2	30.3	543	56	16.7	8.9	125
12	3.00 (1)	100 (1)	30 (0)	34.2	31.5	654	47	18.9	10.6	146
13	1.88 (0)	75 (0)	30 (0)	39.5	46.2	727	41	20.0	11.1	155
14	1.88 (0)	75 (0)	30 (0)	39.7	46.3	725	42	20.1	11.2	155
15	1.88 (0)	75 (0)	30 (0)	39.6	46.1	725	41	20.1	11.0	156

**Table 2:** Regression coefficients calculated for the proposed models for *Mastocarpus stellatus* and the corresponding statistical parameters (99% confidence, *p* < 0.05).

Coefficients	CEY, %	SC, %	G′_o (1 Hz),_ Pa	T_gel_, °C	PC, mg/g	GAE, mg/g	TEAC, mg/g
a_0_	−32.3 ^a^	−29.8 ^a^	−663.4 ^a^	156.9 ^a^	−4.75 ^a^	−3.84 ^a^	−74.4 ^a^
a_1_	20.3 ^a^	21.6 ^a^	454.9 ^a^	−38.1 ^a^	7.91 ^a^	4.94 ^a^	72.3 ^a^
a_2_	0.63	0.8	9.70 ^a^	−0.68 ^a^	0.17	0.097	1.53 ^a^
a_3_	1.52 ^a^	1.9 ^a^	31.0 ^a^	−2.88 ^a^	0.56 ^a^	0.32 ^a^	5.03 ^a^
a_4_	−0.001	−0.002	−0.015	0.002	−0.001	−0.0005	−0.007
a_5_	−0.008	−0.009	−0.28	0.009	−0.004	−0.004	−0.089
a_6_	0.012	0.014	−1.33 ^a^	0.18 ^a^	−0.028 ^a^	−0.004	−0.089
a_7_	−4.80 ^a^	−5.50 ^a^	−92.3 ^a^	7.51 ^a^	−1.59 ^a^	−1.01 ^a^	−15.2 ^a^
a_8_	−0.004	−0.006	−0.05 ^a^	0.004	−0.001	−0.0004	−0.007
a_9_	−0.021 ^a^	−0.026 ^a^	−0.39 ^a^	0.033 ^a^	−0.006 ^a^	−0.004 ^a^	−0.059 ^a^
F	15.6	6.1	12.1	8.5	5.2	4.3	3.9
R^2^	98.5	95.8	98.4	96.5	96.1	95.3	94.9

^a^ Coefficients significant at *p* > 99%.

**Table 3:** Cytotoxicity for different carrageenan types and tumoral cells lines.

Seaweed	Extraction, Degradation Conditions SOLVENT, SLR, Temp, Time	Antiproliferative Activity Cell Line, IC_50_ (μg/mL)	Reference
κ-carrageenan	Commercial, treated with NaOH and KCl, SLR 1 g/100 mL + 0.1 M HCl, 60 °C, 4 h	HeLa, 500	[38]
		BGC, 500	
*Eucheuma denticulatum* κ-carrageenan	Commercial, degraded 0.1 M HCl, 60 °C, 4 h	Caco-2, 280	[39]
		HepG2, 450	
*Eucheuma denticulatum* ι-carrageenan	Commercial, degraded 0.1 M HCl, 60 °C, 4 h	Caco-2, 1000	[39]
*Kappaphycus alvarezii* κ-carrageenan	Distilled water SLR 1 g/35 mL, 2 h + boiling, 30 min + NaOH, boiling, 4 h	HT-29, 73.87	[40]
		HepG2 56.71	
		MG63, 47.85	
κ-carrageenan	Commercial	HT-29, 123.8 Hep G2, 125.0	[40]
		MG63, 55.48	
*Laurencia papillosa* κ-, ι-, λ-carrageenan	Water, 90 °C, 12 h, solvent purification	MCF-7, 200, 50, 25 µM	[11]
*Eucheuma spinosum* ι-carrageenan	Commercial	LM2, 200	[5]
*Hypnea muscifomis* κ-carrageenan	Water, room temp., 24 h + Water, 90 °C, 8 h 0.1 M trifluoroacetic acid, 80 °C, 0.5–3 h	LM2, 100–250	[5]
*Iridaea undulosa* κ/ι-hybrid carrageenan	Water, SLR 2 g/100 mL, room temp., 16 h, alkaline 0.1 M trifluoroacetic acid, 80 °C, 0.5–3 h	LM2, 150–200	[5]
*Gigartina pistillata* λ/ξ hybrid carrageenan	Acetone:methanol (1:1), 16 h, 4 °C, 1 M NaOH, SLR: 1 g/150 mL, 85–90 °C, 3 h	HT-29, 0.705	[3]
*Gigartina pistillata* κ/ι hybrid carrageenan	Acetone:methanol (1:1), 16 h, 4 °C, 1 M NaOH, SLR: 1 g/150 mL, 85–90 °C, 3 h	HT-29, 0.657	[3]
*Mastocarpus stellatus* κ/ι hybrid carrageenan	UAE (80 kHz, 79% amplitude): Water, 2.1 g/100 g, 80 °C, 36.5 min	A-549, 7.5	This work
		A-2780, 7.2	This work
		HeLa-229, 51.9	This work
		HT-29, 36.5	This work

Human cancer cell lines: A-2780 (ovarian carcinoma); A-549 (lung adenocarcinoma); MG63 (osteosarcoma); Caco-2 (epithelial colorectal adenorcarcinoma); HeLa-229 (cervix carcinoma); HepG2 (human hepatocellular carcinoma); HT-29 (colon carcinoma); MCF-7 (breast cancer); MG63 (osteosarcoma); BGC (gastric carcinoma); carcinoma (Hela) Murine cancer cell lines: LM2 (mammary adenocarcinoma). *IC_50, cyclophosphamide, HT-29_* = 44.33 µg/mL; *IC_50, cyclophosphamide, Hep G2_* = 27.55 µg/mL; *IC_50, cyclophosphamide, MG63_* = 26.48 µg/mL; *IC_50, Tamoxifen, Caco-2_* = 7 µg/mL; *IC_50, Tamoxifen, Hep G2_* = 6 µg/mL; *IC_50, Salinomycin, HT-29_* = 0.145 µg/mL; *IC_50, Cisplatin, A-2870_* = 0.39 µg/mL; *IC_50, Cisplatin, HT-29_* = 11.4 µg/mL; *IC_50, Cisplatin, Hela-229_* = 0.92 µg/mL; *IC_50, Cisplatin, A-549_* = 7.46 µg/mL.

## Data Availability

Data are contained within the article.
